# NR4A3 regulates anoikis resistance and metastasis of bladder cancer through EWSR1

**DOI:** 10.1080/15384047.2025.2535774

**Published:** 2025-08-05

**Authors:** Li Fan, Yulin Zhou, Shouyong Liu, Xinfeng Zhuo, Le Qu, Ding Wu, Suchun Wang, Xin Pan, Tangliang Zhao, Feng Xu, Jingping Ge, Wenquan Zhou

**Affiliations:** a Department of Urology, Jinling Hospital, Affiliated Hospital of Medical School, Nanjing University, Nanjing, Jiangsu, China; b Outpatient Department, Jinling Hospital, Affiliated Hospital of Medical School, Nanjing University, Nanjing, Jiangsu, China

**Keywords:** Bladder cancer, NR4A3, endoplasmic reticulum stress, anoikis, EWSR1

## Abstract

Bladder cancer (BLCA) is a common urinary malignancy with high metastatic potential. However, the mechanisms underlying its progression remain unclear. This study aimed to investigate the role and regulatory mechanisms of NR4A3, a nuclear receptor involved in apoptosis and tumor suppression, in BLCA progression, particularly its impact on anoikis resistance and metastasis. NR4A3 expression levels were analyzed using the GEPIA database. Functional studies were conducted by overexpressing NR4A3 in adherent and suspension-cultured BLCA cells. Apoptosis, invasion, migration, and ER stress marker (Bip and CHOP) expression were evaluated. Subcutaneous and lung metastasis models in BALB/c nude mice were used for in vivo validation. GEPIA analysis showed that NR4A3 is significantly downregulated in BLCA. NR4A3 overexpression increased apoptosis, reduced invasion and migration, and upregulated Bip and CHOP expression. *In vivo*, NR4A3 overexpression significantly reduced lung metastasis in BALB/c nude mice (*n* = 8 per group, *p* < .001). Mechanistically, NR4A3 promoted ER stress by regulating the EWSR1/Ezrin pathway, thereby suppressing anoikis resistance. NR4A3 functions as a tumor suppressor in BLCA by enhancing endoplasmic reticulum stress and inhibiting anoikis resistance through the EWSR1/Ezrin pathway. It may serve as a promising therapeutic target for metastatic BLCA.

## 1. Introduction

Bladder cancer (BLCA) is the most frequent malignant tumor of the urinary system. In 2022, there were 613,800 new cases worldwide, ranking ninth.^1^ About 25% of diagnosed BLCA patients have muscular invasive bladder cancer, which has a higher tendency to metastasize to lymph nodes and other organs, and the 5-year survival rate is about 60–70%.^2,3^ The clinical treatment of BLCA is transurethral resection of the tumor combined with intravesical infusion of chemotherapy drugs.^4^ However, the tumor recurrence rate was as high as 40%-80% one year after surgery, and about 50% of patients still developed distal metastasis after treatment.^4^ Hence, the intensive research of the pathogenesis of BLCA and seeking for potential targets is the key to cure BLCA and inhibit its metastasis.

Tumor metastasis is a highly complex and multi-step process, including detachment from the primary tumor, invasion into surrounding tissues, migration through the circulatory or lymphatic system, and eventual adhesion and colonization at distant sites.^5^ A critical barrier to this process is anoikis, a form of programmed cell death that is triggered when cells lose attachment to the extracellular matrix (ECM) or neighboring cells.^6^ By eliminating detached cells that lack proper anchorage, anoikis serves as a defense mechanism to prevent inappropriate survival and outgrowth in foreign microenvironments, thereby inhibiting metastasis. However, many tumor cells develop the ability to evade anoikis, which is essential for their survival during circulation and for establishing secondary tumors.^7^ In particular, highly malignant tumor cells with strong metastatic potential often exhibit enhanced resistance to anoikis, allowing them to survive detachment, travel through the body, and successfully colonize distant organs.^8^ Thus, resistance to anoikis is now recognized as a key enabler of tumor metastasis.^7^ Studies have shown that tumor cells can resist anoikis in a variety of ways, for example, high expression of certain molecules such as Ras, Raf, NF-kB, etc.; Enhance intracellular oxidative stress level; Epithelial mesenchymal transformation (EMT) activation, and so on, which lead to the activation of cell survival signals and the inhibition of apoptotic pathways, and ultimately leads to anoikis resistance and promotes the metastasis.^9–12^ It has been found that anoikis resistance also exists in BLCA,^13^ but the internal molecular mechanism remains to be elucidated.

Nuclear receptor subfamily 4 group A member 3 (NR4A3), a member of the nuclear receptor superfamily members, also referred to as Nor-1.^14^ It acts as an inhibitor in various tumors, such as lung cancer, liver cancer, leukemia, neuroblastoma and other tumors by triggering cell apoptosis.^15–18^ Studies have indicated that NR4A3, as a tumor suppressor gene in BLCA, restrains the growth and metastasis of BLCA by regulating EMT.^19^ Therefore, we assuming that NR4A3 might be enrolled in the regulation of anoikis resistance in BLCA. However, the mechanism by which NR4A3 inhibits anoikis resistance in BLCA and thus inhibits its progression is still unknown. It has been reported that NR4A1, another member of the same family as NR4A3, can migrate to the endoplasmic reticulum (ER) and bind to the protein Bcl-2/TRAPγ, thereby inducing the outflow of Ca^2+^ and the depletion of calcium pool in the ER, followed by ER stress and apoptosis.^20^ Therefore, we speculated that NR4A3 might also inhibit the anoikis resistance of BLCA cells by promoting ER stress, thus inhibiting the metastasis of BLCA.

Emerging evidence has highlighted the pivotal role of the EWSR1/Ezrin signaling axis in tumor progression and metastasis. EWSR1 (Ewing sarcoma breakpoint region 1) is an RNA-binding protein that, when fused with transcription factors like FLI1, forms oncogenic fusion proteins implicated in various sarcomas.^21^ Ezrin, a member of the ERM (Ezrin-Radixin-Moesin) family, functions as a membrane-cytoskeleton linker and is involved in cell shape, adhesion, and motility. In Ewing sarcoma cells, the EWSR1-FLI1 fusion protein upregulates Ezrin expression, leading to enhanced phosphorylation of focal adhesion kinase (FAK) at tyrosine 397, thereby promoting cell migration and invasion.^22^ Although the role of Ezrin in BLCA is less well-characterized, studies have shown that Ezrin expression correlates with tumor grade and may serve as an independent predictor of muscle-invasive disease.^23–25^ These findings suggest that the EWSR1/Ezrin axis could contribute to anoikis resistance and metastatic potential in BLCA, warranting further investigation into its mechanistic involvement.

Based on these observations, we hypothesized that NR4A3 may act as a negative regulator of anoikis resistance in BLCA by inducing ER stress, thereby suppressing metastatic progression. To test this hypothesis, we first examined the clinical correlation between NR4A3 expression and BLCA patient prognosis. We then performed functional and mechanistic studies using both in vitro and in vivo models to evaluate the role of NR4A3 in regulating anoikis resistance and metastasis through ER stress pathways. This study aims to uncover a novel function of NR4A3 in the context of BLCA progression, providing new insights into the molecular mechanisms underlying anoikis resistance and identifying potential therapeutic strategies for metastatic BLCA.

## 2. Methods

### 2.1. Analysis of the expression of NR4A3 and its correlation with prognosis of BLCA

Expression and survival data for NR4A3 in bladder cancer (BLCA) were retrieved from the GEPIA database (http://gepia.cancer-pku.cn/). Differential gene expression between tumor (*n* = 404) and normal tissues (*n* = 28) was analyzed using |log₂ fold-change| >1 and q-value <0.01 as filtering thresholds. The relationship between NR4A3 expression and TNM stage was assessed using the chi-square test. For survival analysis, patients were divided into high and low expression groups based on the median expression value (GEPIA default cutoff). Kaplan – Meier survival curves for overall survival (OS) and disease-free survival (DFS) were constructed, and statistical significance between groups was evaluated using the log-rank test.

### 2.2. Clinical samples

A total of 40 tumor tissues and 40 paired adjacent normal tissues were collected from patients diagnosed with BLCA who underwent surgery at Jinling Hospital were collected between January 2020 and December 2022. Adjacent normal tissues were defined as non-tumorous bladder tissues located at least 2 cm away from the tumor site, obtained from the same patient, and confirmed to be histologically normal by two independent pathologists. The expression level of NR4A3 in these tissues was evaluated by immumohistochemical staining. This study was approved by the ethics committee of the hospital (Approval No. 2023DZKY–037–01). All patients signed informed consent, and all procedures involving human samples were conducted in accordance with the Declaration of Helsinki.

### 2.3. Cell culture

Four BLCA cell lines 5637, RT4, T24 and UMUC3, and a human ureteral epithelial immortalized cell line SV-HUC-1 were purchased from the cell Bank of the Chinese Academy of Sciences. These two cell lines were adhesive cultured with McCoy’s 5a medium (Sigma Aldrich, Steinheim, Germany; Cat. No. M8403) and RPMI-1640 medium (Sigma Aldrich, Steinheim, Germany; Cat. No. R8758,), respectively, added with 10% fetal bovine serum (Corning, NY, USA; Cat. No. 35–011-CV) and 1% Penicillin-Streptomycin solution (Yaji, Shanghai, China; Cat. No. YC18012). Subsequently, BLCA cells were cultured in suspension to simulate the environment of anoikis of tumor cells *in vitro*. Cells were cultured in suspension when they were growing to logarithmic phase or the confluence reaches about 90%. After the cells were resuspended with 0.25% trypsin, they were inoculated into 6-well plates pre-treated with poly-HEMA (Sigma Aldrich, Steinheim, Germany; Cat. No. P3932) to prevent cell adhesion, with 1 × 10^5^ cells/well and 2–3 mL of complete medium added to suspend the cells. Then, they were cultured in a constant temperature cell incubator at 37°C with 5% CO_2_, and subsequent experiments were carried out after 24 h.

### 2.4. NR4A3 overexpression vector construction and infection

Recombinant adenoviral vectors carrying the full-length coding sequences (CDS) of human NR4A3 (NM_006981.3) and EWSR1 (NM_001163287.1), designated as ad-NR4A3 and ad-EWSR1, respectively, were constructed by Hanbio Co., Ltd. (Shanghai, China) using standard cloning procedures. An adenovirus expressing a non-targeting sequence was used as the negative control (ad-NC). Adenovirus infection was performed by referring to the guideline. In short, 1.5 × 10^8^ pfu/mL of ad-NC or ad-NR4A3/ad-EWSR1 was diluted in serum-free medium. Next, T24 or 5637 cells were treated with the diluted adenovirus. After 12 h, the supernatant was discarded and replaced with corresponding medium without Penicillin-Streptomycin and cultured for another 12 h. After 24 h of infection, the expression level of NR4A3 and EWSR1 was examined by Real-time quantitative PCR (RT-qPCR).

### 2.5. Cell transfection

SiRNAs against NR4A3 and EWSR1 (si-NR4A3 and si-EWSR1) and their negative control si-NC were synthesized by Genomeditech (Shanghai, China). They were transferred into T24 and 5637 cells by using Lipofectamine 3000 reagent (Thermo Fisher, Waltham, USA; Cat. No. L3000015). The transfection reagent and siRNAs were diluted with Opti-MEM medium (Gibco, Carlsbad CA, USA; Cat. No. 31985070), the final concentration of siRNA was 50 nM. Then they were mixed and injected into the cell culture medium for transfection. The efficiency of the transfection was validated by RT-qPCR. The sequences of siRNAs were listed in Table S1.

### 2.6. RT-qPCR

Trizol (Psaitong, Beijing, China; Cat. No. PS0808) was used to extract RNA from T24 and 5637 cells. Subsequently, the cDNA was prepared using the extracted RNA and the One-Tube ds-cDNA Synthesis Kit (Yeasen, Shanghai, China; Cat. No. ZY-130308). Then the One Step RT-qPCR Probe Kit (Yuchun Biology, Shanghai, China; Cat. No. 11145ES50) was used to prepare the RT-qPCR reaction system, the LC480 II Real-Time PCR instrument (Roche, Rotkreuz, Switzerland) was applied to perform the reaction. The PCR reactions were carried out in a total volume of 20 μL, containing 10 μL SYBR Mix, 0.4 μL forward and reverse primers (10 μM each), 1 μL cDNA, and 8.2 μL nuclease-free water. The primers used in this research were shown in Table S2.

The cycling conditions were as follows: Initial denaturation at 95°C for 30 s, followed by 40 cycles of: Denaturation at 95°C for 10 s, annealing/extension at 60°C for 30 s. Melting curve analysis was performed from 65°C to 95°C to confirm amplification specificity.

### 2.7. Flow cytometry

The cell apoptosis was detected by flow cytometry. Briefly, 4T1 or 5637 cells were collected and the density was adjusted to 1 × 10^6^/mL using pre-cooled PBS. The cells were then fixed with the cool alcohol for 1 h and filtered with 400 mesh strainers to obtain single-cell suspension. Subsequently, 5 µL PI solution was added and stained in dark for 30 min. Finally, a flow cytometer (BD Accuri C6, USA) was used to monitor the apoptosis rate.

### 2.8. Western blot

Cells were lysed and centrifuged to obtain the supernatant. The protein concentration was quantified by BCA kit (BioVision, San Francisco, USA; Cat. No. K814–5000). Then SDS-PAGE was conducted to separate the proteins and transfer assay was carried out subsequently. Whereafter, the PVDF membranes (Millipore, MA, USA; Cat. No. ISEQ00010) were blocked with 5% BSA solution. Afterward, the membranes were immersed in the diluted primary antibodies at 4°C overnight. The second day, the PVDF membranes were rinsed and continue reacted with secondary antibodies. Finally, ECL luminescence reagent (Sangon Biotech, Shanghai, China; Cat. No. C500044–0100) was dropped onto the PVDF membranes and the bands were visualized and recorded using a chemiluminescence imager (Azure C280, CA, USA). The antibodies used here were exhibited in Table S3.

### 2.9. Transwell assay

This experiment was conducted to evaluate the migration and invasion capacity of T24 and 5637 cells. Transwell chambers (Corning, NY, USA; Cat. No. CLS3422-48EA) containing 8 μm polycarbonate membrane were used. The experimental procedure of migration and invasion assay is similar, expect that for the assessment of invasion, the chambers need to be coated with Matrigel (BD Biosciences, MA, USA; Cat. No. 356237) one day in advance. Briefly, medium containing FBS was pre-injected into 24-well plates, then 2 × 10^5^ cells were spread in the upper cavity and cultured for 24 h or 48 h. Finally, migrating or invading cells were stained with 0.1% crystal violet (Acmec, Shanghai, China; Cat. No. AC11528) and imaged under a microscope.

### 2.10. Animal experiment

BALB/c nude mice (male, 6 weeks old, 18–20 g) were obtained from Enhancer-Bio (Nanjing, China) and randomly assigned to ad-NC and ad-NR4A3 groups (*n* = 8). All animal experiments were reviewed and approved by the Animal Ethics Committee of Jinling Hospital (Approval No. 2023JLHGZRDWLS–00065) and carried out in accordance with the ARRIVE guidelines and institutional regulations for the care and use of laboratory animals. NR4A3-overexpressed T24 cells or cells infected with ad-NC were collected and washed. Then 1 × 10^6^ cells were suspended in 200 mL saline solution and injected into mice via the tail vein. Tumor volume was measured every 3 days using a digital caliper. The volume was calculated as (length × width^2^/2, where length and width represent the longest and shortest tumor diameters, respectively. All measurements were performed by investigators blinded to group allocation. After 4 weeks, the In Vivo Imaging System (IVIS Spectrum, PerkinElmer, USA) was used for photographing. After sacrificing, the lungs were collected for further tests.

### 2.11. Histological staining

The collected lung tissues were immersed in 4% paraformaldehyde solution (Solarbio, Beijing, China; Cat. No. P1110) for 24 h, then the tissues were embedded with paraffin and sliced. For H&E staining, the slices were dewaxed, and the nuclei and cytoplasm were stained with hematoxylin and eosin staining solution (Regal, Shanghai, China; Cat. No. XT0255) in turn. After dehydration and transparency, the slices were sealed and the lung metastasis of BLCA cells was observed with the microscope (Nikon Eclipse E100, Japan). For IHC staining, 3% H_2_O_2_ aqueous solution (Sigma-Aldrich, MO, USA; Cat. No. 88597) was dropped to the dewaxed slices and incubated with the slices at room temperature for 10 min. After cleaning with PBS for 3 times, slices were boiled in 1 × sodium citrate solution (Mackin, Shanghai, China; Cat. No. S885300) for 3 min to repair the antigen. After cooled naturally, the slices were incubated with 10% BSA. Next, the slices were reacted with 1:500 diluted anti-NR4A3 (GTX78011, GeneTex, US), 1: 1000 diluted anti-Bip (3177, Cell signaling technology, MA, USA) or 1: 200 diluted anti-CHOP (bs-1361 R, Bioss, Beijing, China) overnight at 4°C. The following day, after rinsing with PBS and sucking up the residual liquid, the secondary antibody (1: 500, BL003A, Biosharp, Anhui, China) was added and incubated with the slices for 30 min. After rinsing, DAB solution (Boster, Hubei, China; Cat. No. SA2025) was added and developed for 5 min. Afterward, the slices were re-stained using hematoxylin (Solarbio, Beijing, China; Cat. No. G1080). Finally, after rinsing with PBS, the sections were dehydrated, permeabilized, sealed, and observed with the microscope.

IHC staining was semi-quantitatively evaluated using the H-score method, which combines staining intensity and the percentage of positive tumor cells. Staining intensity was scored as follows: 0 (negative), 1 (weak), 2 (moderate), and 3 (strong). The H-score was calculated as: H-score = Σ (percentage × intensity), yielding a range from 0 to 300. Patients were categorized into high and low NR4A3 expression groups based on the median H-score.

### 2.12. TUNEL staining

This experiment was performed to visualize the apoptosis in situ. The TUNEL Apoptosis Detection Kit (Yeasen, Shanghai, China; Cat. No. 40307ES) was used. Briefly, tissue sections were dewaxed, hydrated and permeabilized by protease K. Afterward, TUNEL reaction mixture were dropped onto the tissue sections in a wet box and kept at 37°C for 2 h. Later, the DAPI dye (Sangon, Shanghai, China; Cat. No. E607303) was introduced and stained for 30 min away from light. After rinsing, the sections were observed and recorded with the fluorescence microscope (Nikon Eclipse 80i, Japan).

### 2.13. Chromatin immunoprecipitation (ChIP) assay

Firstly, BLCA cells were cross-linked and immobilized with 1% formaldehyde (Sigma Aldrich, Steinheim, Germany; Cat. No. F1635), then terminated with 0.125 M glycine. Subsequently, the cells were rinsed with ChIP-buffer and ultrasonicated for 20 min (The ultrasonic condition was 45% power, 10 s off and 10 s on). The homogenate was centrifugated at 8000×g for 10 min at 4°C, then the supernatant was immunoprecipitated with antibodies on Protein-A/G-Sepharose beads (Abcam, Cambridge, UK; Cat. No. Ab193262). After washing, elution and de-cross-linking, the ChIP DNA was verified by PCR.

### 2.14. Detection of EWSR1–FLI1 fusion transcript

Total RNA was extracted from BLCA cells, and cDNA was synthesized as described in the RT-qPCR section above. RT-PCR was conducted using primers specific to the EWSR1–FLI1 fusion transcript. The EWSR1–FLI1 fusion transcript sequence used in this study corresponds to the Type I fusion (EWSR1 exon 7–FLI1 exon 6). The amplified product was verified by Sanger sequencing. The obtained sequences were analyzed using SnapGene software and aligned to reference sequences (EWSR1: NM_005243.4; FLI1: NM_002017.5) via the NCBI BLAST tool to confirm the fusion junction. The primer sequences were provided in Table S2.

### 2.15. Statistical analysis

Data were exhibited as the means ± SEM and analyzed using software SPSS 23.0. Student’s *t*-test was applied to detect the difference between two groups. ANOVA was applied to compare the differences between multiple groups. Kaplan – Meier survival curves for overall survival (OS) and disease-free survival (DFS) were generated using data from the GEPIA database. Patients were divided into high and low expression groups based on the median NR4A3 expression, and survival differences were assessed using the log-rank test. *p* < .05 was set as the threshold.

## 3. Results

### 3.1. NR4A3 is low-expressed in BLCA

**Figure 1.**
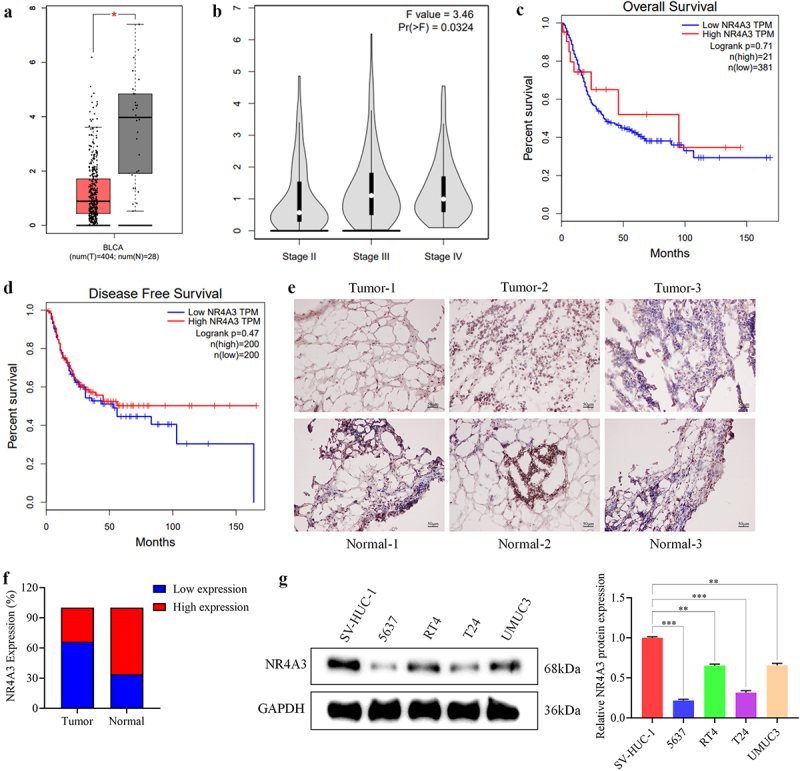
NR4A3 level in BLCA and its relationship with prognosis. (a). NR4A3 was low-expressed in BLCA. (b). Expression levels of NR4A3 in different TNM stages of BLCA. The relationship between NR4A3 level and the overall survival (c) and disease-free survival (d) of BLCA patients. (e). Representative immunohistochemical staining images of NR4A3 in BLCA tumor tissues and adjacent tissues. (f). Statistical graph of the expression level of NR4A3 in tumor and normal tissues. (g). The expression of NR4A3 in four BLCA cell lines and human ureteral epithelial immortalized cell line was evaluated by Western blot. *p < 0.05, **p < 0.01, **p < 0.001.

NR4A3 expression in BLCA was analyzed through the GEPIA website. NR4A3 expression was significantly lower in BLCA tissues compared to normal tissues, with a log₂ fold-change of −0.95 and *p* < .01, based on GEPIA analysis (Figure 1a). Significant differences were found in the expression level of NR4A3 in different TNM stages of BLCA (Figure 1b). In addition, the survival analysis curves for overall survival (OS) and disease-free survival (DFS) were plotted to investigate the correlation between NR4A3 level and patients’ prognosis. It was confirmed that patients with high level of NR4A3 had higher OS and DFS (Figure 1c,d). In addition, we examined the level of NR4A3 in clinical samples. In the study cohort, among the 40 BLCA samples, 12 cases exhibited high NR4A3 expression and 28 cases showed low expression; among the 40 adjacent tissue samples, 27 cases had high NR4A3 expression and 13 cases had low expression (Figure 1e). The low expression rates of NR4A3 in BLCA tissues and adjacent tissues were 67.5% and 32.5%, respectively. Compared with adjacent tissues, NR4A3 expression was markedly lower in BLCA tissues (Figure 1f). We also found that the low expression of NR4A3 was significantly associated with the pathological stage of BLCA samples and the low survival rate of BLCA patients (Fig. S1). Moreover, we confirmed that NR4A3 was low-expressed in four BLCA cell lines, including 5637, RT4, T24 and UMUC3 (Figure 1g).

### 3.2. NR4A3 inhibits the anoikis resistance and metastasis ability of BLCA cells

**Figure 2.**
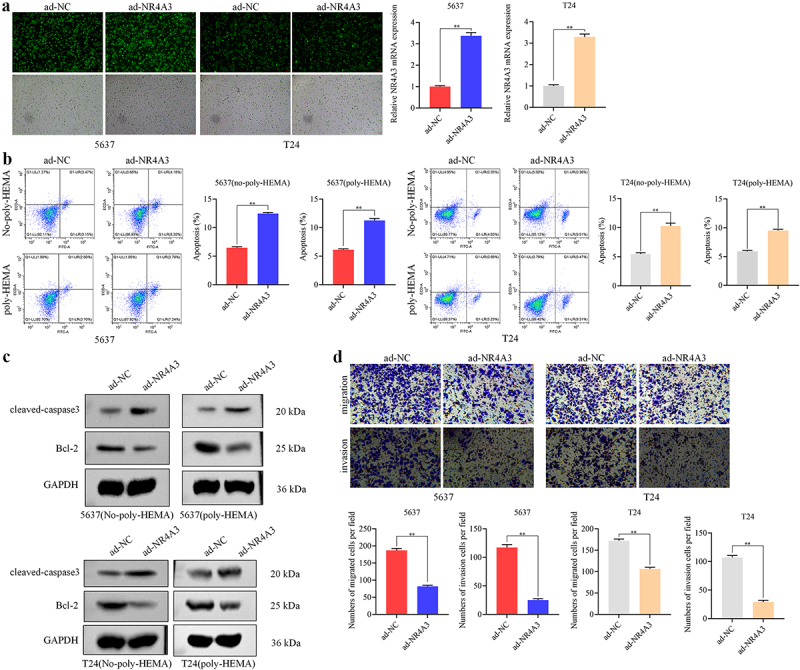
NR4A3 inhibits the anoikis resistance and metastasis ability of BLCA cells. (a). The infection efficiency of ad-NR4A3 and ad-NC in 5637 and T24 cells was verified by detecting GFP fluorescence and RT-qPCR assay. Scale bar = 100 μm. The infected 5637 and T24 cells were treated with or without poly-HEMA, the apoptosis rate and the expression of Cleaved-caspase3 and Bcl-2 were determined by flow cytometry (b) and Western blot (c), respectively. (d). The migration and invasion ability of the infected cells were detected by transwell assay. Scale bar = 50 μm. **p < 0.01.

To clarify the role of NR4A3 in BLCA anoikis resistance and metastasis, adenovirus expression vectors were used to mediate NR4A3 overexpression in T24 and 5637 cells, the efficiency of infection was validated by the GFP fluorescence in cells and the RT-qPCR, the mRNA expression of NR4A3 was elevated by more than twofold (Figure 2a). Fig. S5 shows representative qPCR amplification curve and melting curve. The apoptotic rate of T24 and 5637 cells overexpressing NR4A3 was found significantly increased with or without treatment with poly-HEMA (*p* < .01, Figure 2b). Moreover, the expression of Cleaved-caspase3 was increased and Bcl-2 was decreased in both T24 and 5637 cells treated with or without poly-HEMA (Figure 2c). Furthermore, we demonstrated that overexpression of NR4A3 reduced the migration and invasion ability of T24 and 5637 cells (*p* < .01, Figure 2d). Additionally, we demonstrated that the knockdown of NR4A3 in RT4 cells further suppresses apoptosis while enhancing their migration and invasion (Fig. S2). These findings suggest that NR4A3 inhibits the anoikis resistance and metastasis ability of BLCA cells.

### 3.3. NR4A3 inhibits anoikis resistance by promoting ER stress

**Figure 3.**
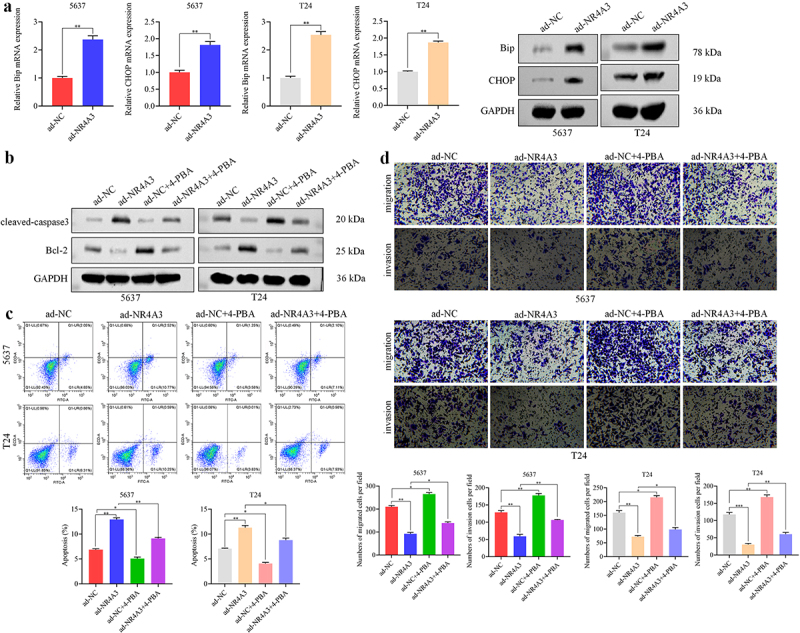
NR4A3 inhibits anoikis resistance by promoting ER stress. (a). The mRNA and protein expressions of Bip and CHOP were evaluated through RT-qPCR and Western blot. The infected 5637 and T24 cells were treated with or without 4-PBA, and the expression of Cleaved-caspase3 and Bcl-2 was determined by Western blot (b), the apoptosis rate was detected by flow cytometry (c), and the migration and invasion were detected by transwell assay (d). Scale bar = 50 μm. *p < 0.05, **p < 0.01.

Next, we investigate the mechanism by which NR4A3 suppresses anoikis resistance in BLCA cells. The mRNA and protein levels of Bip and CHOP, which were related to ER stress, were evaluated. RT-qPCR analysis indicated that compared with the control group, overexpression of NR4A3 increased the expression of CHOP by approximately 0.75 times and the level of Bip by approximately 1.5 times in 5637 and T24 cells (*p* < .01); Western blot analysis also confirmed this (Figure 3a). Subsequently, 4-PBA was used to inhibit the occurrence of ER stress in cells. We observed that BLCA cells overexpressing NR4A3, when pretreated with 4-PBA, exhibited a reduction in Cleaved-Caspase 3 expression, an increase in Bcl-2 expression (Figure 3b), a lower apoptosis level (Figure 3c), and enhanced migration and invasion (Figure 3d) compared to the ad-NR4A3 group. Furthermore, we evaluated the oxidative stress levels in 5637 cells following NR4A3 overexpression. The results indicated that while there was a slight increase in oxidative stress in the ad-NR4A3 group, this difference was not statistically significant compared with the ad-NC group (Fig. S3). The above results indicate that NR4A3 suppresses the anoikis resistance, migration and invasion of BLCA cells by promoting ER stress rather than other factors such as oxidative stress.

### 3.4. *Overexpression of NR4A3 restrains the growth and metastasis of BLCA* in vivo

**Figure 4.**
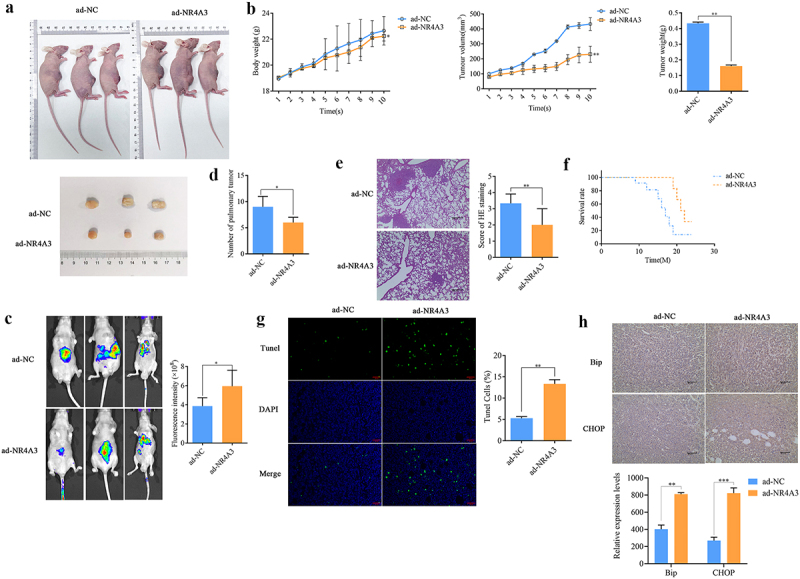
Overexpression of NR4A3 inhibits the growth and metastasis of BLCA in vivo. (a). Images of subcutaneous xenograft tumor model nude mice injected with ad-NC and ad-NR4A3 and the isolated tumor. Arrows indicate the location of the tumors. (b). Tumor weight and volume of the nude mice in ad-NC and ad-NR4A3 group. (c). in vivo imaging of lung metastases in two groups of nude mice. (d). Number of pulmonary tumor in nude mice. (e). HE staining of lung tissue slices of mice in two groups. Scale bar = 200 μm. (f). The statistics of survival of two groups of mice. TUNEL staining (g, scale bar = 20 μm) and immunohistochemical staining (h, scale bar = 50 μm) of the tumors. *p < .05, **p < .01.

Although our research shows that over-expressed NR4A3 confines the anoikis resistance, migration and invasion of BLCA cells, it is still unknown whether NR4A3 can inhibit the malignant progress of BLCA *in vivo*. Therefore, we constructed BLCA subcutaneous xenotransplant tumor model and lung metastasis model for further exploration. The representative images of mice on the Day 10 indicated that overexpressed NR4A3 inhibits tumor growth (Figure 4a). Tumor growth was monitored over 4 weeks, revealing a significant difference starting at Day 10. By Week 4, tumors in the NR4A3-overexpression group were 100% smaller than in controls (*p* < .01) and the body weight also slightly decreased (*p* < .05) (Figure 4b). Then the *in vivo* imaging was performed on lung metastasis model mice, the images showed that overexpression of NR4A3 reduced the lung metastasis of BLCA (*p* < .05, Figure 4c). After the nude mice were killed and dissected, it was found that the overexpression of NR4A3 reduced the number of pulmonary tumor (*p* < .05, Figure 4d), and the HE staining of lung tissues also confirmed that the overexpression of NR4A3 reduced the lung metastasis of BLCA (Figure 4e). Furthermore, it was confirmed that overexpression of NR4A3 was beneficial to improve the survival of nude mice bearing tumors (Figure 4f). In addition, the effect of NR4A3 overexpression on the anoikis and ER stress of BLCA *in vivo* was explored. TUNEL staining results indicated that NR4A3 overexpression increased the apoptotic rate of BLCA tissues (*p* < .01, Figure 4g). At the meantime, the expression of Bip and CHOP in tumor tissues was increased by 1-fold and 2.2-fold, respectively (Figure 4h). These results indicated that the overexpression of NR4A3 *in vivo* inhibited the growth and lung metastasis of BLCA, and inhibited the anoikis resistance by promoting ER stress.

### 3.5. NR4A3 regulates the EWSR1/Ezrin pathway in BLCA

**Figure 5.**
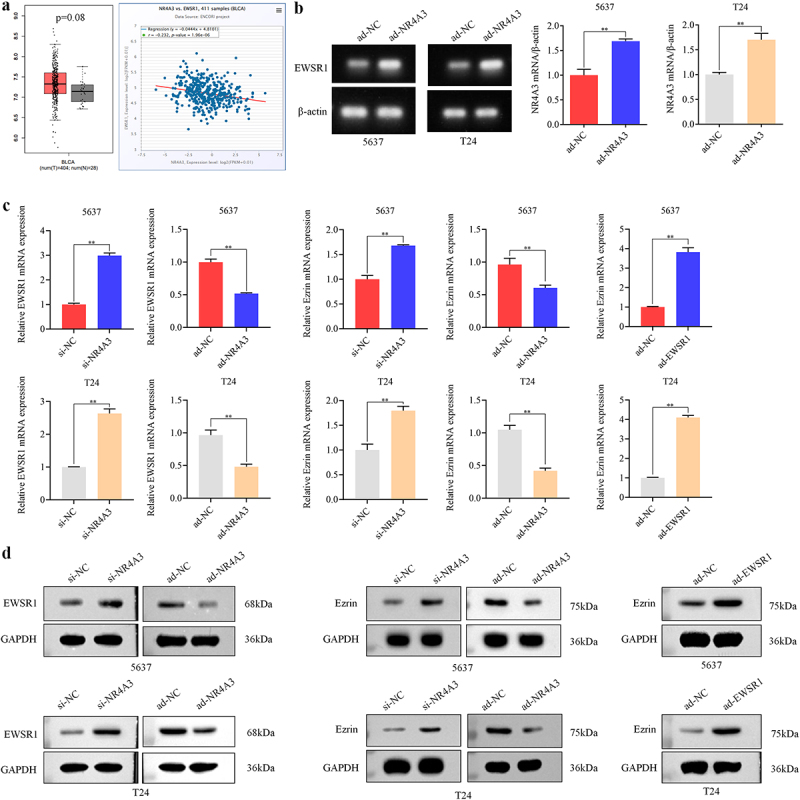
NR4A3 regulates the EWSR1/Ezrin pathway in BLCA. (a). The expression level of EWSR1 in 404 BLCA tumor tissues and 28 adjacent normal tissues from TCGA database was analyzed. The correlation between EWSR1 and NR4A3 expression levels in BLCA clinical samples was analyzed. (b). The protein level of EWSR1 in 5637 and T24 cells that infected with ad-NR4A3/ad-NC was determined by Western blot. The mRNA and protein levels of EWSR1, Ezrin in 5637 and T24 cells that overexpressing NR4A3 or knocking down NR4A3, and Ezrin in 5637 and T24 cells that overexpressing EWSR1, were detected by RT-qPCR (c) and Western blot (d), respectively. **p < 0.01.

**Figure 6.**
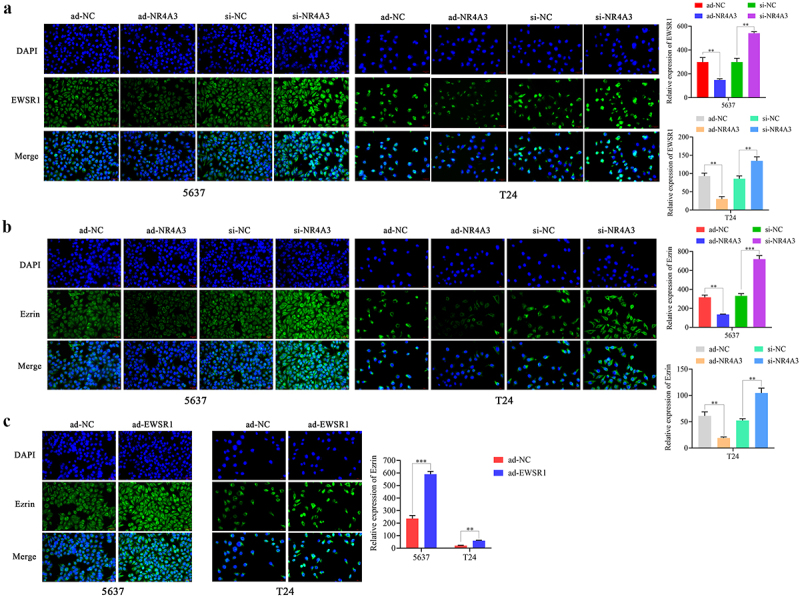
The expression of EWSR1 (a), Ezrin (b) in 5637 and T24 cells that overexpressing NR4A3 or knocking down NR4A3, and Ezrin in 5637 and T24 cells that overexpressing EWSR1 (c), were visualized by immunofluorescent staining. Scale bar = 20 μm. **p < 0.01, ***p < 0.001.

EWSR1 has been proven to be associated with the migration of anoikis cells in Ewing’s sarcoma. Next, we explored the regulatory role of NR4A3 on EWSR1/Ezrin pathway in BLCA. Firstly, GEPIA website was employed to analyze the level of EWSR1 in BLCA samples. The expression of EWSR1 was found increased in BLCA, and was negatively related to the expression of NR4A3 (Figure 5a). Through ChIP assay, it was confirmed that NR4A3 could directly bind to the promoter of EWSR1 (Figure 5b). Furthermore, through RT-qPCR, Western blotting and Immunofluorescence staining assays, we demonstrated that decreased NR4A3 promoted the expression of EWSR1 and Ezrin, while overexpression of NR4A3 inhibited the expression of EWSR1 and Ezrin. In NR4A3 overexpressing 5637 and T24 cells, the overexpression of EWSR1 promoted the level of Ezrin (Figures 5c,d and 6). EWSR1 usually forms a fusion gene with FLI1 and expresses a fusion protein, which becomes a carcinogenic driver factor. To confirm the presence of the EWSR1–FLI1 fusion transcript, we performed RT-PCR and Sanger sequencing using fusion-specific primers. The sequencing confirmed the junction of EWSR1 exon 7 and FLI1 exon 6 (Supplementary Fig. S4), indicating the expression of a Type I fusion transcript. These findings indicated that NR4A3 regulates the EWSR1/Ezrin pathway in BLCA.

### 3.6. NR4A3 affects the ER stress by regulating EWSR1/Ezrin pathway and thus interferes with anoikis resistance and metastasis

**Figure 7.**
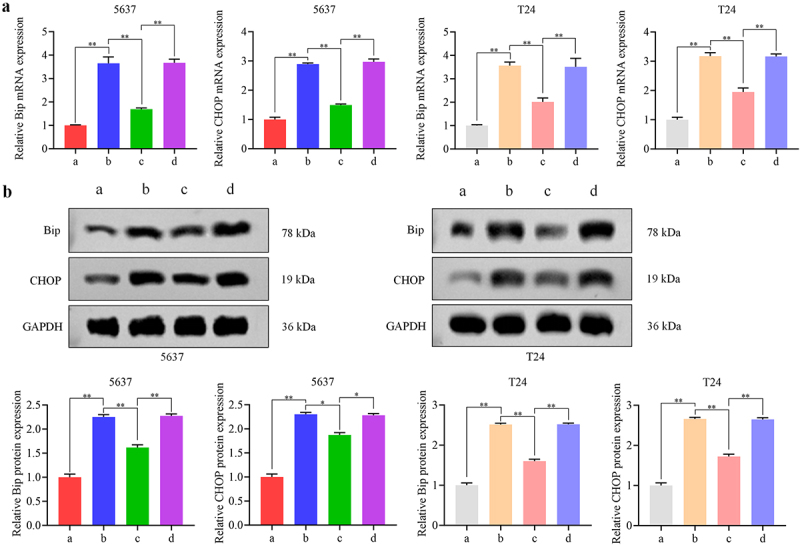
NR4A3 affects the ER stress by regulating EWSR1/Ezrin pathway. 5637 and T24 cells were divided into four groups: a: ad-NC + ad-NC + si-NC, b: ad-NR4A3 + ad-NC + si-NC, c: ad-NR4A3 + ad-EWSR1 + si-NC, and d: ad-NR4A3 + ad-EWSR1 + si-Ezrin group. The relative mRNA expression and protein expression levels of Bip and CHOP in 5637 and T24 cells in these four groups were assessed by RT-qPCR (a) and Western blot (b). *p < 0.05, **p < 0.01.

**Figure 8.**
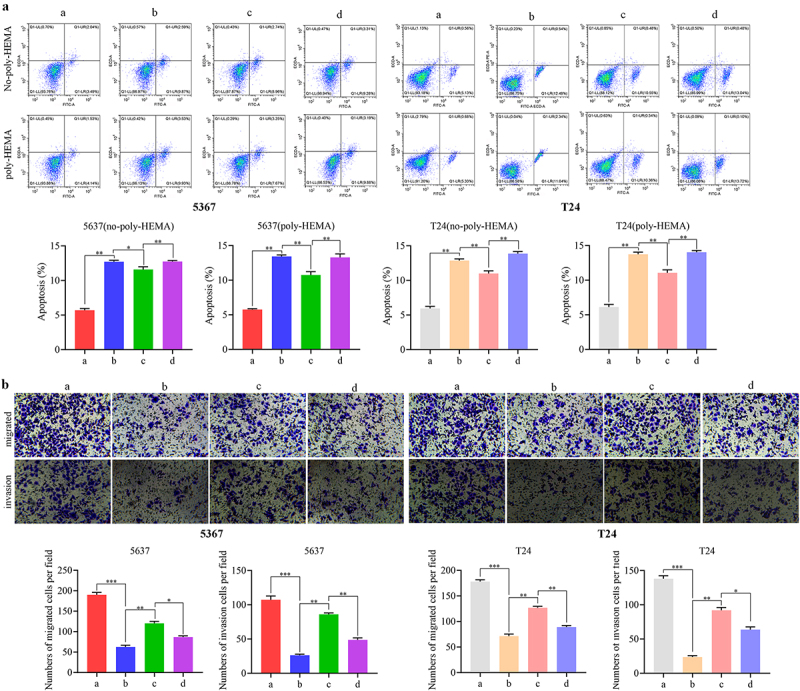
NR4A3 affects the ER stress by regulating EWSR1/Ezrin pathway and thus interferes with anoikis resistance and metastasis. 5637 and T24 cells were divided into four groups: a: ad-NC + ad-NC + si-NC, b: ad-NR4A3 + ad-NC + si-NC, c: ad-NR4A3 + ad-EWSR1 + si-NC, and d: ad-NR4A3 + ad-EWSR1 + si-Ezrin group. (a). Cell apoptosis of cells treated with or without poly-HEMA was detected. (b). The migration and invasion of cells in these four groups were detected by transwell assay. Scale bar = 50 μm. *p < 0.05, **p < 0.01, **p < 0.001.

To clarify whether NR4A3 regulates anoikis resistance and metastasis of BLCA by affecting ER stress through regulating the EWSR1/Ezrin pathway, we overexpressed NR4A3 and/or EWSR1, and/or silenced Ezrin. BLCA cells were divided into four groups: ad-NC + ad-NC + si-NC, ad-NR4A3 + ad-NC + si-NC, ad-NR4A3 + ad-EWSR1 + si-NC, and ad-NR4A3 + ad-EWSR1 + si-Ezrin group. Dramatically, the mRNA and protein expression of Bip and CHOP in the ad-NR4A3 + ad-NC + si-NC group was increased, and their expression was significantly decreased after overexpression of EWSR1. When knocking down Ezrin, the level of Bip and CHOP was obviously enhanced. These trends were the same in 5637 and T24 cells (Figure 7a,b). Flow cytometry results showed that overexpression of NR4A3 promoted cell apoptosis with or without poly-HEMA treatment, while apoptosis was significantly inhibited by co-overexpression of EWSR1, and further knockdown of Ezrin aggravated the apoptosis (Figure 8a). Moreover, it was confirmed by Transwell assay that overexpression of NR4A3 inhibited the cell migration and invasion, co-overexpression of EWSR1 significantly promoted this ability of BLCA cells; while simultaneously knocking down Ezrin partially inhibited the migration and invasion ability of BLCA cells (Figure 8b). These findings confirmed that NR4A3 could affect ER stress by regulating EWSR1/Ezrin pathway, thereby interfering with anoikis resistance and metastasis of BLCA.

**Figure 9.**
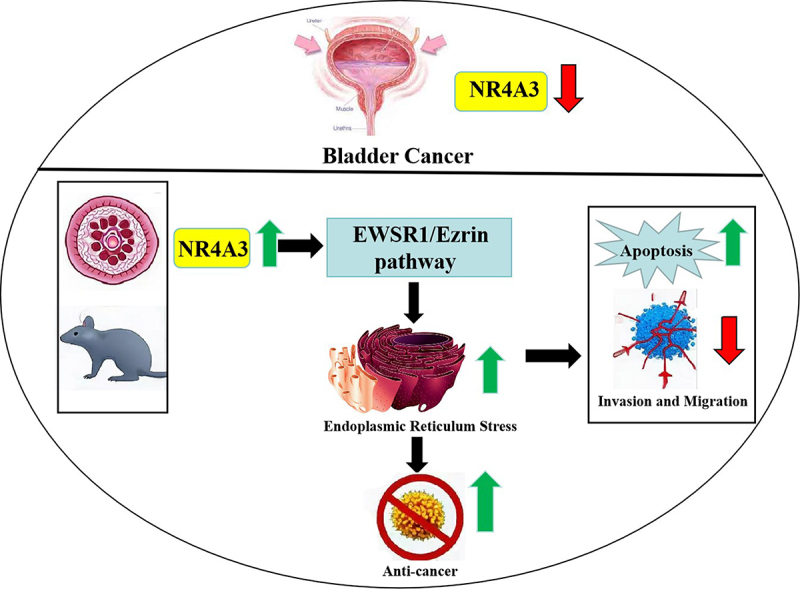
Schematic illustration of the proposed mechanism by which NR4A3 contributes to disease pathogenesis via the EWSR1/Ezrin signaling pathway.

To provide a clearer mechanistic understanding of how NR4A3 deficiency contributes to pathogenesis via the EWSR1/Ezrin pathway, and how gene restoration may offer therapeutic potential, we have included an illustrative schematic (Figure 9).

## 4. Discussion

NR4A3 has been recognized as a tumor suppressor in various tumors.^26^ Consistent with this, our study revealed that NR4A3 is significantly downregulated in bladder cancer (BLCA) based on GEPIA database analysis, and its lower expression correlates with advanced TNM staging and poor patient survival. These findings suggest a potential tumor-suppressive role of NR4A3 in BLCA; however, its functional mechanisms have remained largely unclear.

Anoikis is a specific type of programmed cell death triggered by the loss of cell – extracellular matrix attachment, and resistance to anoikis is common in tumor cells, enabling them to survive in suspension, facilitating metastasis and invasion.^27^ However, studies on the relationship between NR4A3 and anoikis are scarce. In this study, we mimicked the anoikis environment in vitro by suspending BLCA cell lines T24 and 5637. We found that NR4A3 overexpression significantly promoted apoptosis while inhibiting migration and invasion in both adherent and suspended conditions. These results suggest that NR4A3 inhibits anoikis resistance and metastasis of BLCA cells. Furthermore, using subcutaneous xenografts and lung metastasis mouse models, we demonstrated that NR4A3 overexpression in vivo inhibited tumor growth, reduced lung metastasis, and improved survival. These observations are consistent with findings from other cancers. Deutsch et al. showed that NR4A3 could inhibit aggressive lymphoma by upregulating pro-apoptotic genes,^28^ while Yang et al. found that NR4A3 downregulation enhanced the proliferation and invasiveness of BLCA cells.^19^

ER stress, caused by protein misfolding or accumulation of unfolded proteins, is closely related to tumor progression.^29^ Studies have indicated that ER stress is closely related to the occurrence and development of tumors. On the one hand, ER stress gives the tumor a more powerful tumorigenicity, metastatic ability and drug resistance by affecting the biological function of tumor cells.^30^ At the same time, tumor cells enhance their adaptability through unfolded protein response, and promote tumor progress by regulating the interaction with immune cells.^31^ On the other hand, ER stress can enhance or weaken the anti-tumor immune effect and promote tumor metastasis and therapeutic resistance by affecting the function of immune cells in the tumor microenvironment.^32^ Xia and colleagues suggest that anoikis resistance in hepatocellular carcinoma may depend on ER stress.^11^ Therefore, this study focused on exploring the correlation between NR4A3 and ER stress and anoikis. Here, we treated BLCA cells with poly-HEMA to prevent their adherent growth, thus reducing the contact between cells and the environment and between cells, and finally achieving the purpose of inducing anoikis. We found that overexpression of NR4A3 increased the expression of Bip and CHOP levels under anoikis conditions. Pretreatment with ER stress inhibitor 4-PBA attenuated apoptosis and restored cell migration and invasion capacity, indicating that ER stress mediates the anti-anoikis effect of NR4A3. In vivo results also confirmed that NR4A3 overexpression elevated Bip and CHOP expression in tumor tissues. These findings imply that NR4A3 inhibits anoikis resistance and metastasis of BLCA cells through the induction of ER stress.

The anoikis resistance of tumor cells is regulated by many genes and signal transduction pathways. Previous studies have shown, for example, that TCF7L2 enhances anoikis resistance and metastasis in gastric cancer via PLAUR activation.^33^ Fedorova et al. found that the overexpression of NR4A3 inhibits the proliferation and promotes apoptosis of breast cancer and lung cancer cells by increasing the expression of pro-apoptotic genes PUMA and Bax.^26^ However, there are few studies on the mechanism of anoikis in BLCA, and the molecular mechanism of NR4A3 regulating anoikis in BLCA and influencing the malignant progress of BLCA remains unclear. In this study, we further explored the underlying mechanism in BLCA and found, through GEPIA analysis, that EWSR1 expression was upregulated in BLCA and negatively correlated with NR4A3. This suggested that NR4A3 may regulate BLCA progression via EWSR1. EWSR1 is known to form fusion genes with FLI1 in Ewing sarcoma, where the fusion protein promotes the phosphorylation of tyrosine397 of adhesion kinase by increasing the expression of Ezrin, and ultimately facilitates anoikis resistance and metastasis.^33^ In our study, we confirmed the presence of EWSR1:FLI1 fusion in BLCA cells by Sanger sequencing (Fig. S4), and found that NR4A3 overexpression inhibited the expression of the fusion gene and its downstream target Ezrin. Ezrin, as a linker between membrane and cytoskeleton, promotes adhesion, migration, and metastasis, and has been associated with poor prognosis in multiple cancers.^34–36^ Thus, our findings suggest that NR4A3 may activate ER stress and inhibit anoikis resistance by downregulating the EWSR1–Ezrin axis.

Our study provides new insights into how NR4A3 inhibits malignant progression of BLCA by regulating ER stress and EWSR1. Importantly, it also raises the possibility of targeting NR4A3 as a therapeutic strategy. Although direct activators of NR4A3 are not currently available, potential future approaches may include: (1) using ER stress inducers to amplify NR4A3‘s pro-apoptotic effects; (2) developing gene therapy strategies to restore or enhance NR4A3 expression; and (3) evaluating NR4A3 as a prognostic biomarker in larger BLCA patient cohorts to support its clinical application. These directions could offer novel therapeutic avenues for combating BLCA metastasis.

However, our study also has several limitations. First, although we confirmed the inhibitory effect of NR4A3 on BLCA growth and metastasis in vivo, the downstream molecular mechanism was not validated in animal models. Second, we only investigated the effect of NR4A3 overexpression; loss-of-function studies (e.g., siRNA or CRISPR knockout) are needed to fully establish causality. Finally, although preliminary clinical sample analysis supported the inverse correlation of NR4A3 and EWSR1 in BLCA, large-scale clinical validation is still needed to assess prognostic value and therapeutic applicability.

## 5. Conclusion

In summary, this study demonstrated that low expression of NR4A3 is associated with bladder cancer progression and poor prognosis. Overexpression of NR4A3 promoted ER stress both in vitro and in vivo, thereby suppressing anoikis resistance and metastasis, at least in part through the downregulation of the EWSR1/Ezrin signaling axis. Beyond these findings, future studies should explore whether NR4A3 also participates in regulating chemotherapy resistance or tumor immune response, both of which are critical components of BLCA progression and treatment failure. From a translational perspective, NR4A3 holds potential as a prognostic biomarker, and may be targeted clinically via gene therapy, epigenetic modulation, or pharmacological strategies that enhance ER stress signaling. These directions will help further define the role of NR4A3 in BLCA and support its development as a therapeutic target.

## Supplementary Material

Supplemental Material

## Data Availability

The datasets used and/or analyzed during the current study are available from the corresponding author on reasonable request.
