# Supplementary material for: NR4A3 regulates anoikis resistance and metastasis of bladder cancer through EWSR1
Source: Cancer Biol Ther. 2025 Aug 5;26(1):2535774. doi: 10.1080/15384047.2025.2535774 (PMC12326571; doi:10.1080/15384047.2025.2535774)
Supplement: Supplemental Material [file KCBT_A_2535774_SM7390.zip › SI-Legends.docx]

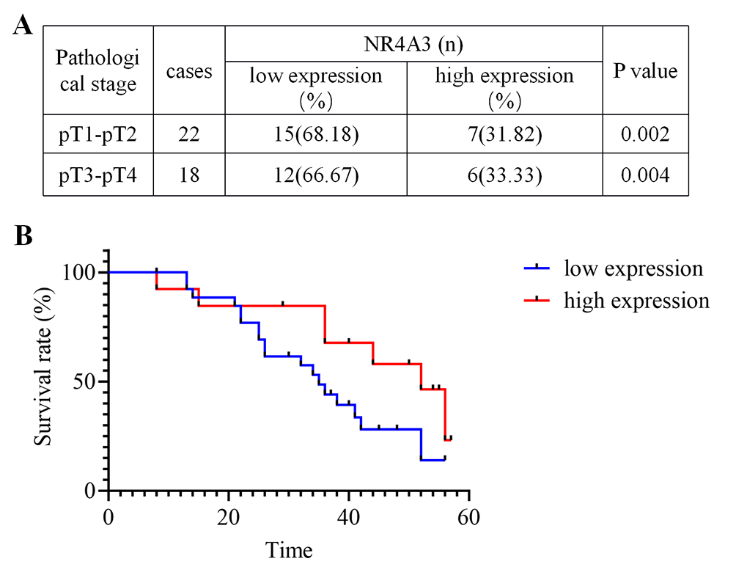


Fig. S1 A. Correlation between the pathological stage of BLCA clinical samples and the expression level of NR4A3. B. The relationship between the expression level of NR4A3 and the postoperative survival rate of patients.


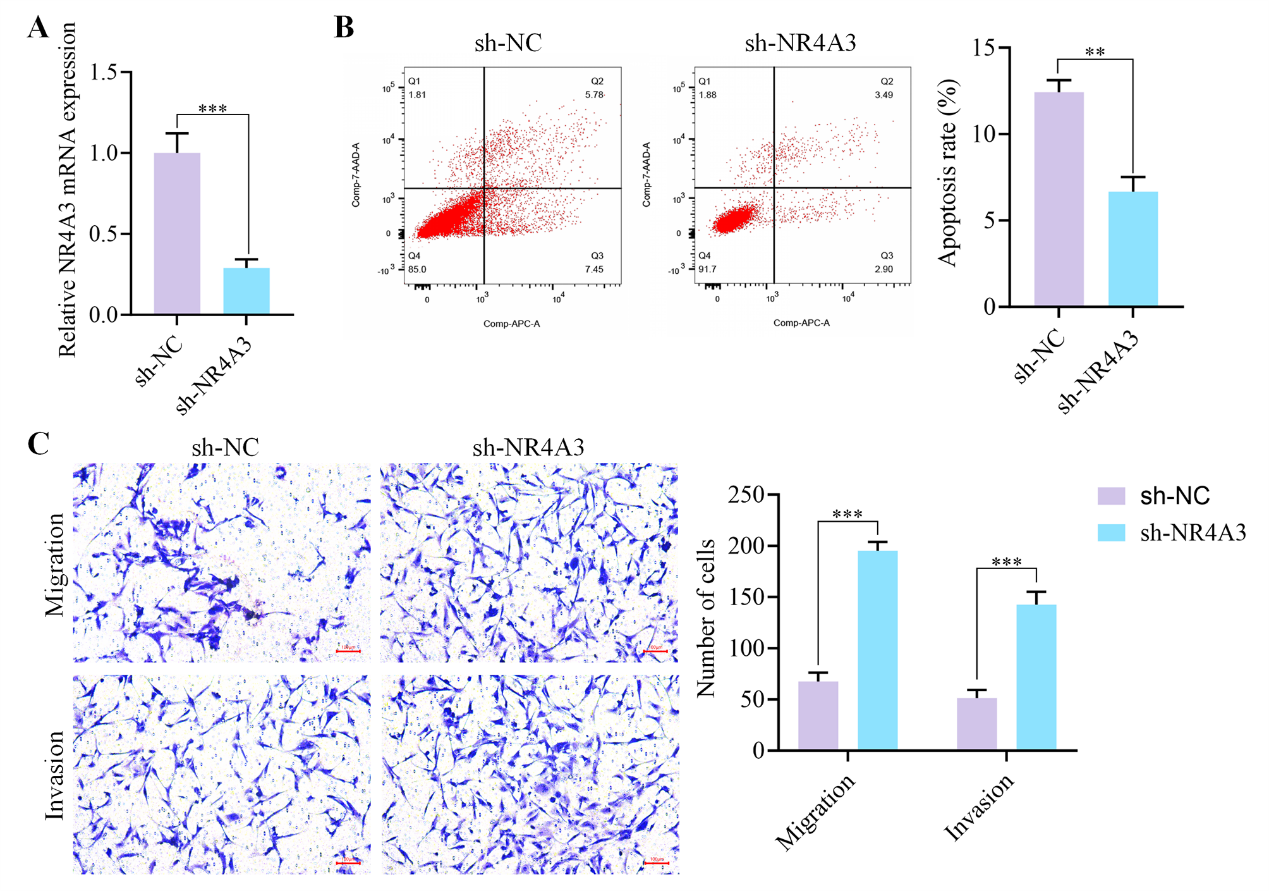


Fig. S2 NR4A3 was knocked down in RT4 cells, and the efficiency was verified by RT-qPCR (A), and cell apoptosis (B), migration and invasion (C) were evaluated.


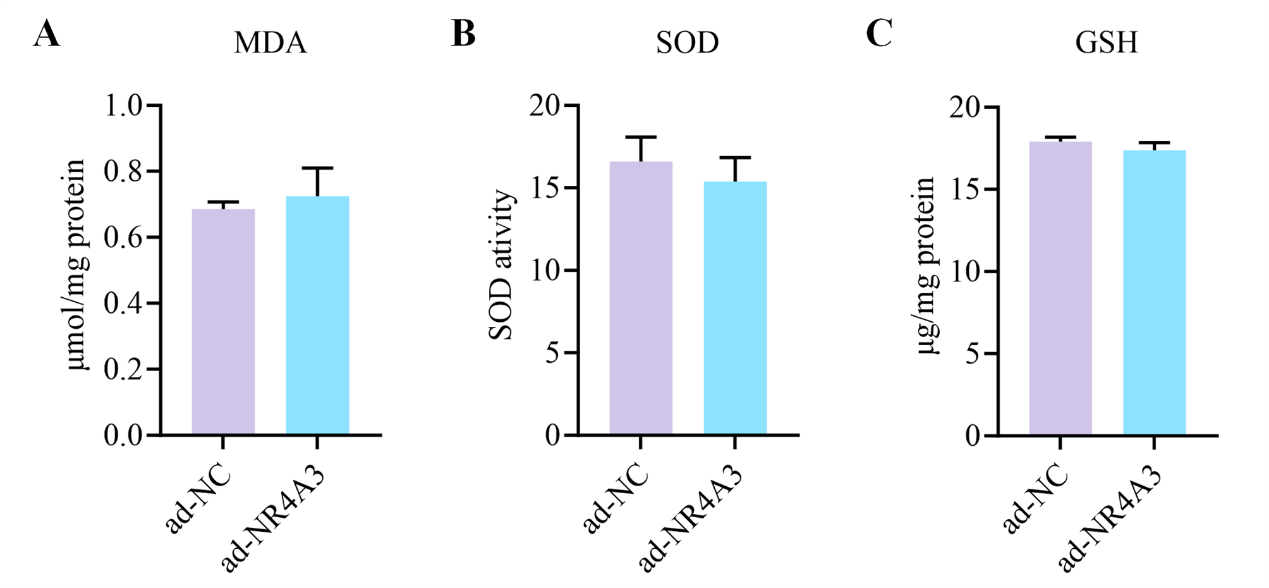


Fig. S3 The oxidative stress levels of 5637 cells after overexpression of NR4A3.


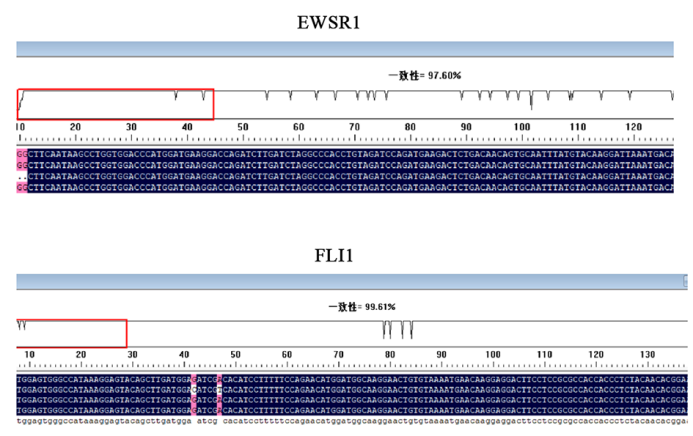


Fig. S4 Sanger sequencing validation of the EWSR1::FLI1 fusion transcript. The circled part in red is the fusion site.


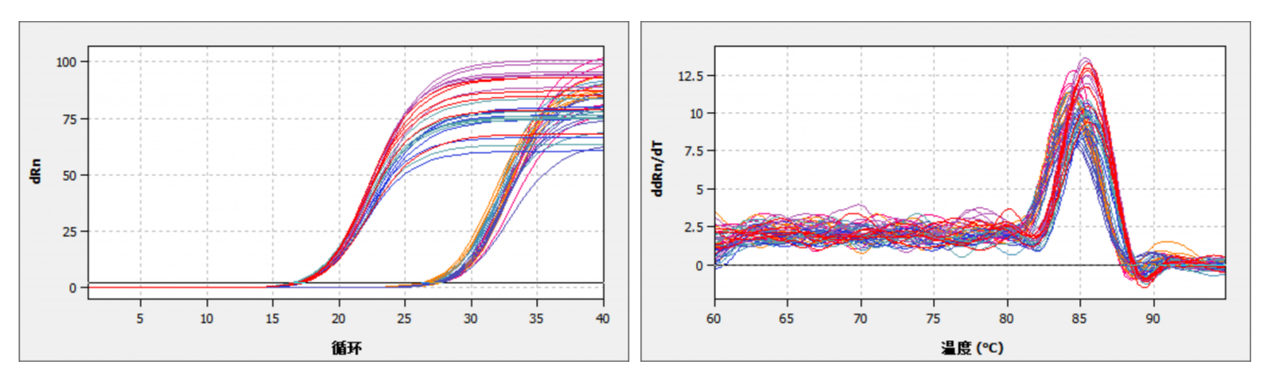


Fig. S5 A representative qPCR amplification curve and melting curve.

**Table S1 Sequences of siRNAs used in cell transfection**

| siRNA | Sequences |
| --- | --- |
| si-NR4A3-1 | 5’-CGCGAGTCACGTACCCAGCAATTAA-3’ |
| si-NR4A3-2 | 5’- GCCAGCATTACGACATCGACTACT-3’ |
| si-NR4A3-3 | 5’- GACCGTATGCGAGTCCAGTACTGCT-3’ |
| si-NC | 5’- CCCATCGTACCTCCCAACCAGATAA-3’ |
| si-EWSR1-1 | 5’-CAAGTCACGATTACAGAACGTC-3’ |
| si-EWSR1-2 | 5’- ACGTCCAAGCAGCTACGCTTGACAG-3’ |
| si-EWSR1-3 | 5’- GACCGATTGATAACGACGTGATG-3’ |
| si-NC | 5’- AACGCCGTACATGACAGTAGCC-3’ |

**Table S2 Primer sequences used in RT-qPCR**

| Gene | Accession number | Primer sequences |
| --- | --- | --- |
| NR4A3 | NM_006981.3 | Forward: 5’-ACTGACCTAAGTACAGCATG-3’  Reverse: 5’-AAGCGTAACTGTTCAGCAG-3’ |
| Bip | NM_005347.7 | Forward: 5’-GACCTAAGACTTGCAGACTG-3’  Reverse: 5’-AAGCGTAACTGTTCAGCAG-3’ |
| CHOP | NM_004083.6 | Forward: 5’-TCGACGATAGCCACCGTACAG-3’  Reverse: 5’-CAGTCCAGTTACGAATCGGC-3’ |
| EWSR1 | NM_005243.4 | Forward: 5’-GACAAGTCTGACGACTAAGC-3’  Reverse: 5’-ATGCACTTGACGACTAGAGCT-3’ |
| Ezrin | NM_003379.5 | Forward: 5’-CAAGTCAATACGACGTTGA-3’  Reverse: 5’-AAGCTAGTTCGAAGCTACAGG-3’ |
| EWSR1-FLI1 fusion | AF043336.1 | Forward: 5’- CAGTCACTGCAGCAGTGCT-3’  Reverse: 5’- TCCTCCACATACAGTCCTTCAG-3’ |
| GAPDH | NM_002046.7 | Forward: 5’-GCACTGATCTGAGTACCTGATAT-3’  Reverse: 5’- GACGTTGCTGATGCGACTTG-3’ |

**Table S3 Information of antibodies used in Western blot assay**

| Antibodies | Catalog# | Dilution rate | Manufacturer |
| --- | --- | --- | --- |
| Cleaved-Capase3 | 9661 | 1:1000 | Cell signaling technology |
| Bcl-2 | 633501 | 1:500 | Biolegend |
| Bip | 3177 | 1:1000 | Cell signaling technology |
| CHOP | 948701 | 1:500 | Biolegend |
| EWSR1 | ab133318 | 1:5000 | Abcam |
| Ezrin | Ab205381 | 1:1000 | Abcam |
| GAPDH | 649201 | 1:500 | Biolegend |
| Goat Anti-Rabbit IgG | ab205718 | 1:20000 | Abcam |
| Goat Anti-Mouse IgG | ab205719 | 1:15000 | Abcam |
