# Supplementary figures and images for: NR4A3 regulates anoikis resistance and metastasis of bladder cancer through EWSR1
Source: Cancer Biol Ther. 2025 Aug 5;26(1):2535774. doi: 10.1080/15384047.2025.2535774 (PMC12326571; doi:10.1080/15384047.2025.2535774)

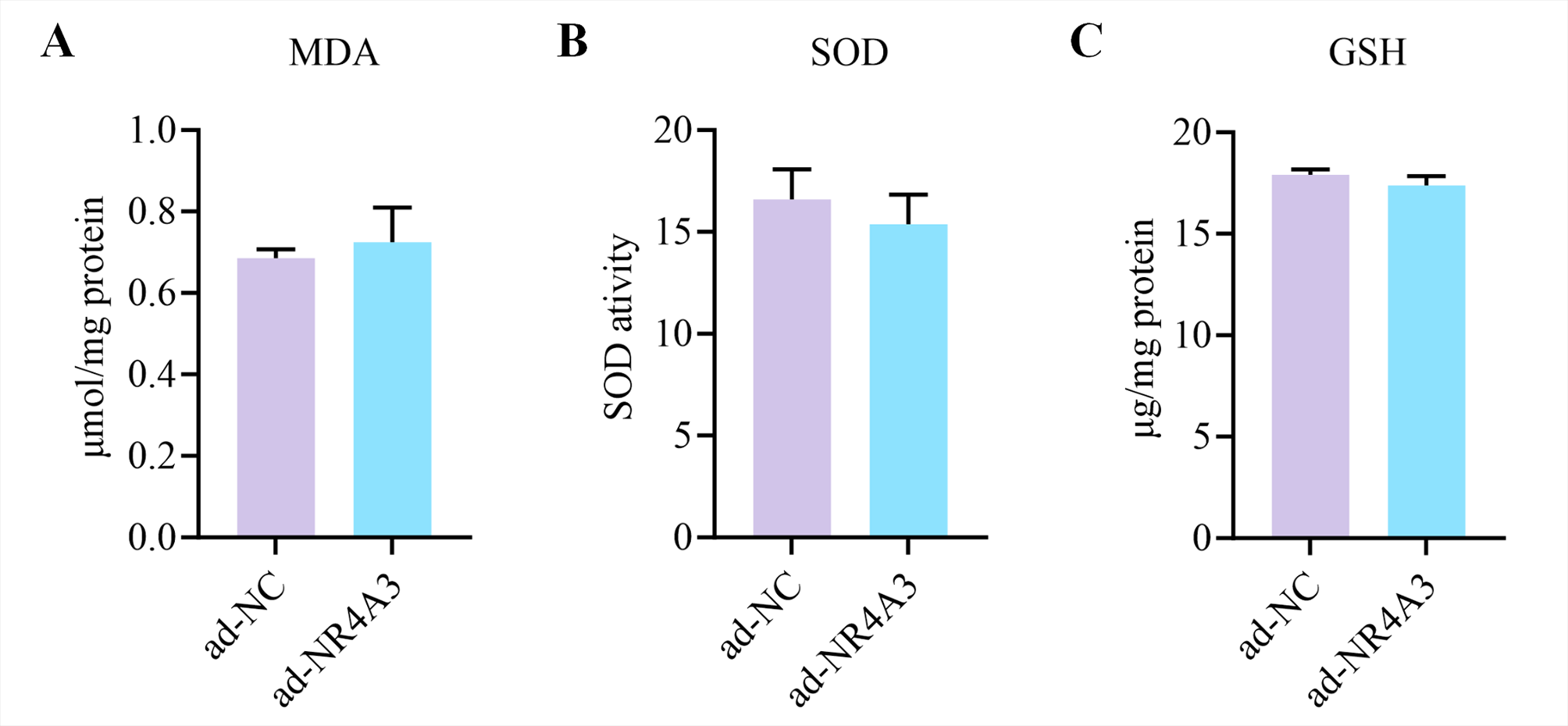

Supplement: Supplemental Material [file KCBT_A_2535774_SM7390.zip › Revised Figure S3.tif]

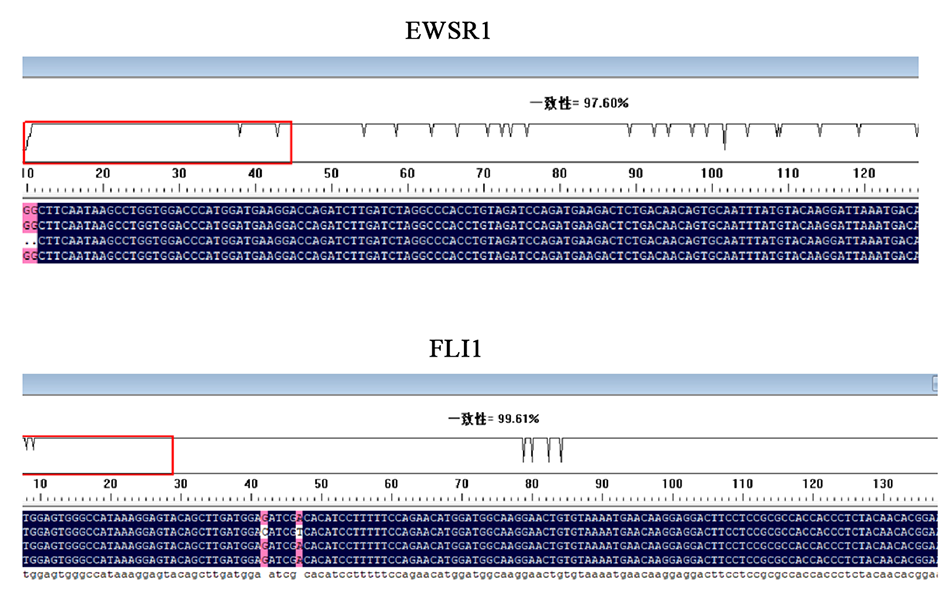

Supplement: Supplemental Material [file KCBT_A_2535774_SM7390.zip › Revised Figure S4.tif]

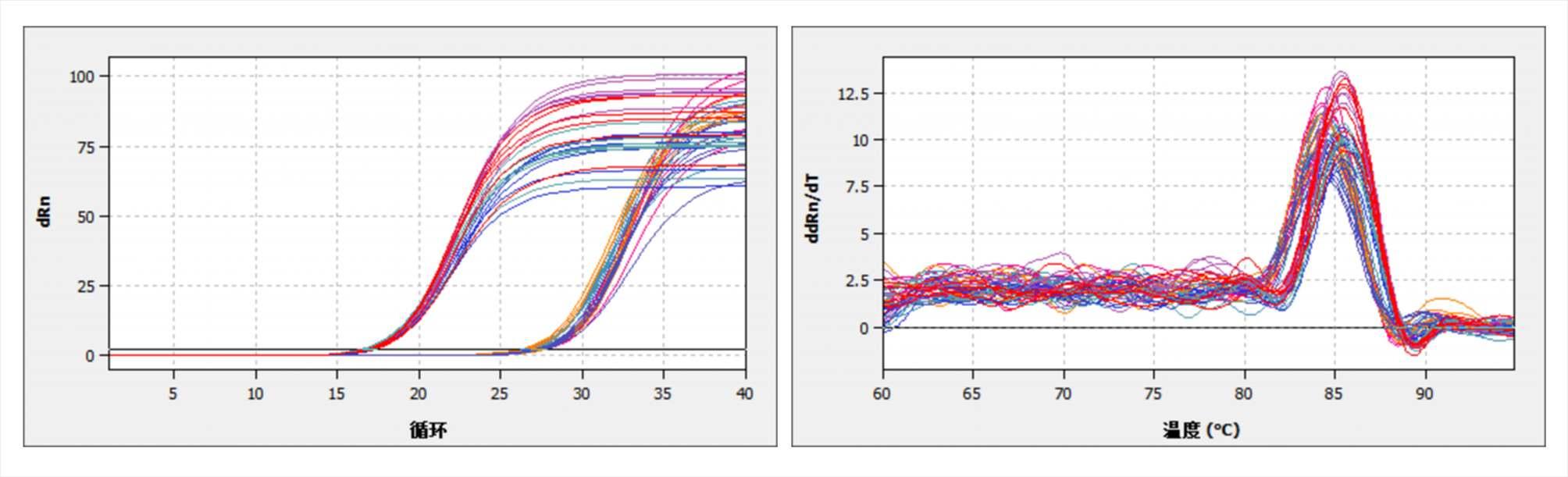

Supplement: Supplemental Material [file KCBT_A_2535774_SM7390.zip › 3rd revised figure S5.tif]

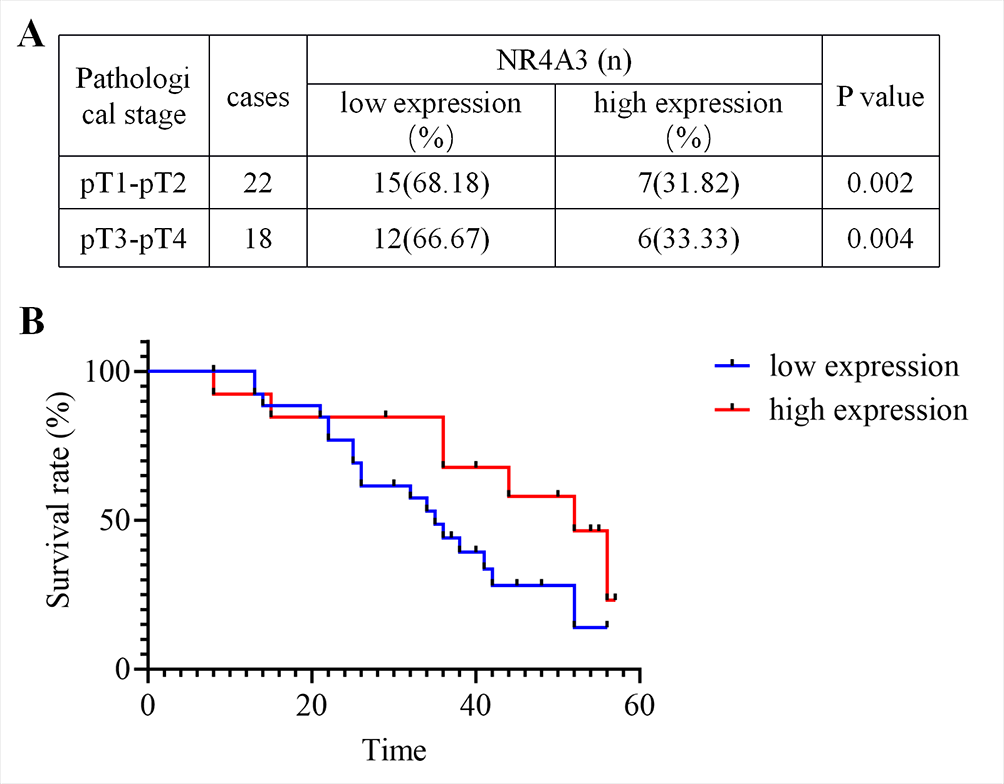

Supplement: Supplemental Material [file KCBT_A_2535774_SM7390.zip › Revised Figure S1.tif]

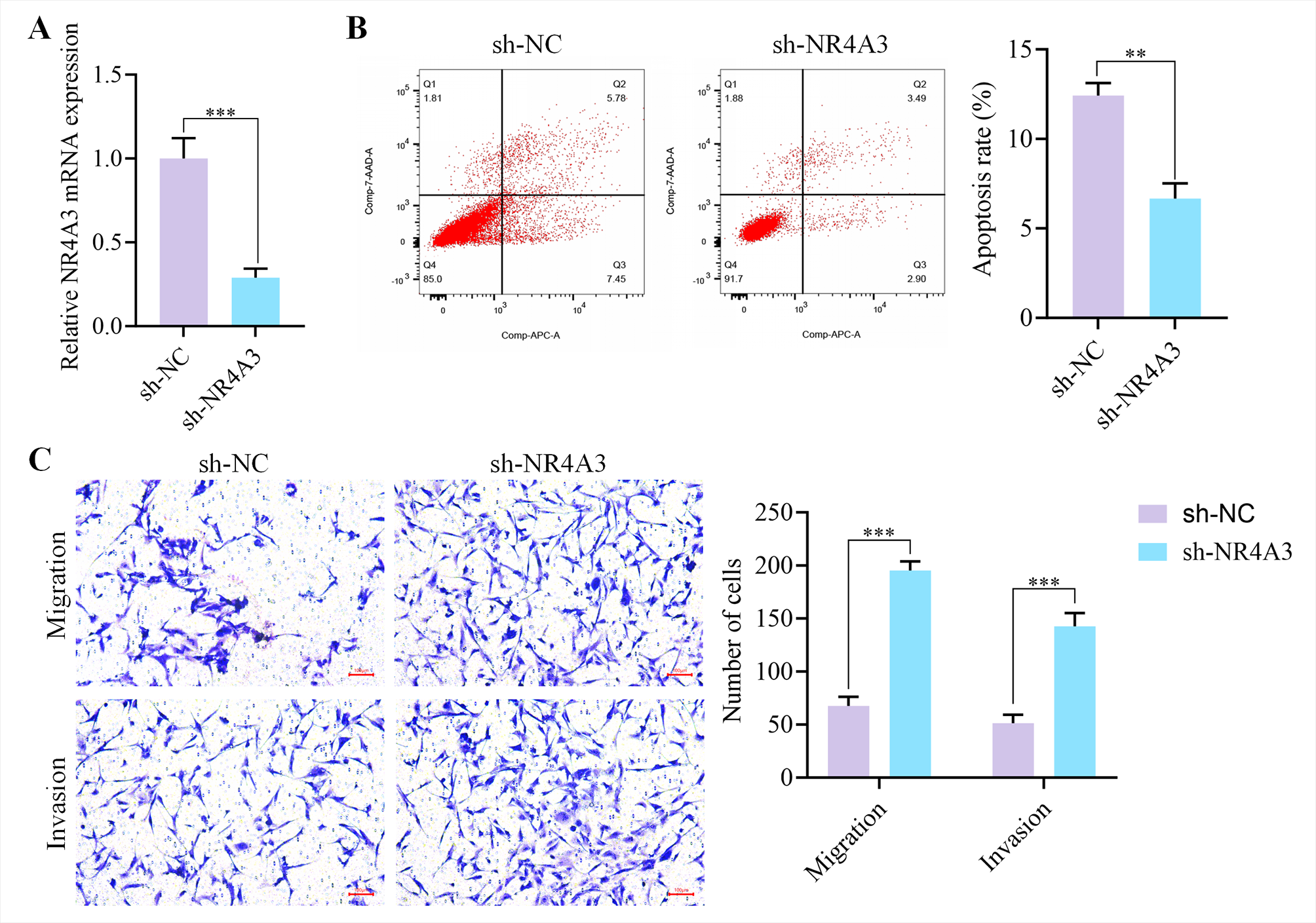

Supplement: Supplemental Material [file KCBT_A_2535774_SM7390.zip › Revised Figure S2.tif]
